# CircZNF609 regulates pulmonary fibrosis via miR-145-5p/KLF4 axis and its translation function

**DOI:** 10.1186/s11658-023-00518-w

**Published:** 2023-12-18

**Authors:** Wenqing Sun, Siyun Zhou, Lan Peng, Yi Liu, Demin Cheng, Yue Wang, Chunhui Ni

**Affiliations:** 1 Department of Occupational Medical and Environmental Health, Key Laboratory of Modern Toxicology of Ministry of Education, Center for Global Health, School of Public Health, Nanjing Medical University, Nanjing, 211166 China; 2 Department of Public Health, Kangda College of Nanjing Medical University, Lianyungang, 320700 China

**Keywords:** Silicosis, circZNF609, Coding capacity, Non-coding RNA

## Abstract

**Background:**

Pulmonary fibrosis is a growing clinical problem that develops as a result of abnormal wound healing, leading to breathlessness, pulmonary dysfunction and ultimately death. However, therapeutic options for pulmonary fibrosis are limited because the underlying pathogenesis remains incompletely understood. Circular RNAs, as key regulators in various diseases, remain poorly understood in pulmonary fibrosis induced by silica.

**Methods:**

We performed studies with fibroblast cell lines and silica-induced mouse pulmonary fibrosis models. The expression of circZNF609, miR-145-5p, and KLF4 was determined by quantitative real-time polymerase chain reaction (qRT-PCR) analysis. RNA immunoprecipitation (RIP) assays and m6A RNA immunoprecipitation assays (MeRIP), Western blotting, immunofluorescence assays, and CCK8 were performed to investigate the role of the circZNF609/miR-145-5p/KLF4 axis and circZNF609-encoded peptides in fibroblast activation.

**Results:**

Our data showed that circZNF609 was downregulated in activated fibroblasts and silica-induced fibrotic mouse lung tissues. Overexpression of circZNF609 could inhibit fibroblast activation induced by transforming growth factor-β1 (TGF-β1). Mechanically, we revealed that circZNF609 regulates pulmonary fibrosis via miR-145-5p/KLF4 axis and circZNF609-encoded peptides. Furthermore, circZNF609 was highly methylated and its expression was controlled by N6-methyladenosine (m6A) modification. Lastly, in vivo studies revealed that overexpression of circZNF609 attenuates silica-induced lung fibrosis in mice.

**Conclusions:**

Our data indicate that circZNF609 is a critical regulator of fibroblast activation and silica-induced lung fibrosis. The circZNF609 and its derived peptides may represent novel promising targets for the treatment of pulmonary fibrosis.

**Supplementary Information:**

The online version contains supplementary material available at 10.1186/s11658-023-00518-w.

## Introduction

Pulmonary fibrosis can have several causes, including ischaemia, infection, autoimmunity, mechanical injury, and occupational and environmental toxins [1]. Silica is one of the most abundant naturally occurring minerals on earth, and occupational exposure to silica dust can lead to silicosis. Silicosis, a potentially fatal lung fibrosis, is a global occupational health issue in contemporary society [2].

In a general scenario, the lung can restore normal organ architecture in response to stimulation and injury; however, dysregulation of this regeneration process can trigger excessive extracellular matrix (ECM) deposition, resulting in lung fibrogenesis and organ dysfunction [2]. The response to inhaled silica particles is typically initiated by alveolar macrophages, which recognize and phagocytose the silica particles, thus inducing the oxidative stress response [2]. Subsequently, polarized macrophages and damaged epithelial cells secret great amounts of inflammatory cytokines and fibrogenic mediators, exacerbating inflammation and leading to fibroblast activation. Fibroblasts are activated and differentiate into myofibroblasts, a core event that occurs in the tissue repair process [3, 4]. The latter secrete excessive ECM and are therefore considered to be the culprits of silica-induced pulmonary fibrosis. Although few antifibrotic therapies are currently available and effective in clinical trials, targeting fibroblasts or myofibroblasts such as inhibiting fibroblast activation and inducing myofibroblast apoptosis, senescence, dedifferentiation, and reprogramming, are still promising potential antifibrotic therapies [5, 6]. Therefore, we are trying to focus on exploring the underlying regulatory molecular mechanisms in fibroblast activation to find potential therapeutic targets.

Circular RNAs (circRNAs) are a kind of unique RNA of exonic or intronic sequences origin and are covalently closed single-stranded RNA molecules [7]. These RNAs were first found in viruses in 1976 and have recently been reported to be abundant in mammalian cells [8]. Mounting evidence has shown that a large number of circRNAs exert significant biological functions via serving as miRNA sponges, interfering with RNA stability and translation, as well as encoding proteins [9, 10]. Considering the well-elucidated roles of circRNAs, researchers have been studying the function of circRNAs in physiology and pathology. Recently, a study showed that some circRNAs were disordered in patients with idiopathic pulmonary fibrosis (IPF), suggesting that circRNAs may act as regulators in the development of pulmonary fibrosis [11]. In addition, recent investigations showed that circRNAs have the ability to be involved in silica-induced pulmonary fibrosis via regulating silica-induced macrophage activation [12] or epithelial-to-mesenchymal transition [13]. Encouraged by these studies, we speculated that circRNAs might also perform an important function in the fibroblast activation stage of silica-induced pulmonary fibrosis.

As mentioned above, circRNAs are the products of back-splicing of precursor mRNA, and thus may share the same type of chemical modifications with mRNA. The N6-methyladenine (m6A) modification is the most common RNA modification [14], and it also has been found in various circRNAs [15]. Lately, researchers have focused on m6A-modified circRNAs and have found that m6A modification regulates the function of circRNAs in various diseases by affecting the circRNA cytoplasmic export, translation, and degradation of circRNAs, etc. [15, 16]. However, the present understanding of m6A modification of circRNAs modification is still the tip of the iceberg, and further research is needed to uncover the underlying mechanisms.

In this study, we found that a specific circRNA, circZNF609, located at chr15:64791491–64792365, was frequently downregulated in lung tissue from silica inhaled mice and in transforming growth factor-β1 (TGF-β1) treated fibroblasts. Functional investigations showed that overexpression of circZNF609 could remarkably inhibit fibroblast activation and silica-induced pulmonary fibrosis in mice. We further revealed that m6A methylation regulates the expression and nuclear-to-cytoplasmic export of circZNF609. Importantly, subsequent experiments showed that circZNF609 could be translated into fibroblasts, indicating that circZNF609 may regulate pulmonary fibrosis via multiple pathways. In summary, this study suggests that circZNF609 functions as a critical regulator in silica-induced pulmonary fibrosis, shedding new light on the development of effective targets for silicosis therapy.

## Materials and methods

### Animal models

Male C57BL/6 mice aged 6 weeks (19–21 g) were obtained from the Animals Core Facility of Nanjing medical university (Nanjing, China). All in vivo experiments were conducted following the agreements authorized by the Laboratory Animal Welfare Ethics Committee of Nanjing Medical University (IACUC-2102038).

Detailed operation procedures of mouse pulmonary fibrosis model induced by silica dust were mentioned previously [17, 18]. Briefly, the anesthetized mice were intratracheally instilled with 0.05 ml sterile saline or silica suspension (50 mg/kg silica particles (Sigma-Aldrich, USA) in 0.05 ml sterile saline). The mice were euthanatized at a certain time (day 7, 14 and 28) and isolated lungs for further study.

For the model of circZNF609 overexpression, the anesthetized mice were intratracheally instilled 50 μl AAV9-circNC/AAV9-circZNF609 per mouse at a titer of 9 × 10^12^ v. g./ml. And after 21 days, mice in each group were treated with the same way of silica suspension as described above. After 28 days, the mice were killed, and the lungs were collected for study.

### Histopathology and hydroxyproline content assay

The fresh lung tissues of mice were fixed by paraformaldehyde. Hematoxylin and eosin (H&E) staining were conducted following standard steps in collaboration with Servicebio Co., Ltd. (Wuhan, China). Collagen degrees were detected using the hydroxyproline assay kit (Jincheng, Nanjing) followed the instructions.

### Cell culture and treatment

MRC-5 cell lines (embryonic lung fibroblasts) were obtained from the Procell (CL-0161, Wuhan, China). The MRC-5 cells were cultured in Minimum Essential Medium (MEM, VivaCell Biosciences, Shanghai, China), and the culture media were added 10% fetal calf serum (VivaCell Biosciences, Shanghai, China) and 1% penicillin–streptomycin (Life Technologies/Gibco, Gaithersburg, MD). Recombinant TGF-β1 (Peprotect, USA) were used to stimulate fibroblast activation.

### Cell transfection

CircZNF609 plasmid, miR-145-5p mimic, miR-145-5p inhibitor, and KLF4 siRNA were purchased from GenePharm (Shanghai, China). CircZNF609-3 × Flag/circZNF609-3 × Flag-mut plasmids were designed by Shanghai Genechem Co., Ltd. (Shanghai, China). Transfection of siRNAs was conducted using riboFECT™ CP Reagent (Ribobio, Guangzhou, China) based on protocols. Plasmid transfection was performed using jetOPTIMUS^®^ transfection reagent (Strasbourg, France) instead.

### Real-time PCR (RT-PCR) and quantitative real-time PCR (qRT-PCR)

500 ng of total RNA was used to synthesis cDNA via HiScript II Q Select RT SuperMix for qPCR (Vazyme Biotech, Nanjing, China). For quantitative real-time PCR, SYBR Green 2 × PCR mix (Vazyme Biotech, Nanjing, China) were used to conduct the amplification reactions according to protocols. For real-time PCR, the cDNA and gDNA PCR products were presented using 2% agarose gel electrophoresis via BIO-RAD GelDoc 2000 (Hercules, CA, USA).

### Fluorescence in situ hybridization assay (FISH)

Cy3-modified circZNF609 probe and FISH probe reaction buffer (F16501/50) were synthesized from GenePharm (Shanghai, China). Fixed MRC-5 cells by 4% paraformaldehyde were incubated with buffer A for 15 min at room temperature and then washed in PBS, treated with 2 × buffer C for 30 min at 37 °C. The circZNF609 probe was diluted with buffer E to 4 μM and denaturized for 5 min at 100 °C. After discarding 2 × buffer C, the cells were hybridized with 100 μl circZNF609 probe mixture at 37 °C overnight. After being washed with 0.1% buffer F, 2 × buffer C, and 1 × buffer C, the cells were sealed with 4′, 6-diamidino-2-pheny-lindole (DAPI, Sigma) for 15 min at room temperature. The whole experiment should be conduct in dark. The images were captured by Fluoview 300 confocal laser scanning microscopy (Olympus, Tokyo, Japan).

### Separation of nuclear and cytoplasmic RNA

Using a PARIS™ Kit (Invitrogen, NY, USA) with the instructions, RNA of MRC-5 cells can be isolated from separate nuclear and cytoplasmic fractions.

### RNA immunoprecipitation (RIP)

Detailed operation steps of RIP assays were described in our previous publication [19].

### Western blot analysis

T-PER Tissue Protein Extraction Reagent (Thermo Scientific) was used to extract tissue protein, while total cell protein was isolated via RIPA buffer (Beyotime, China). Protein quantification and SDS-PAGE gel electrophoresis were executed as previously described [20]. Antibodies for collagen I, fibronectin, α-SMA and KLF4 were buy from Abcam. Antibody for Vimentin was purchased from Cell Signaling Technology. Anti-GAPDH was obtained from ABclonal. The Western blots were visualized and quantified using Image J. The target proteins were normalized with GAPDH.

### Immunofluorescence assay

Fixed MRC-5 cells were blocked with 10% goat serum (Beyotime, China) for 1 h at room temperature, incubated with primary antibody at 4 °C overnight, reacted with Cy3-conjugated secondary antibody (Beyotime, China) for 1 h at room temperature, dyed the nuclei with DAPI (Beyotime, China). Wash with PBST three times for 5 min between each step. The images were acquired by Fluoview 300 confocal laser scanning microscopy (Olympus, Tokyo, Japan).

### EdU fluorescence staining and CCK-8 analysis

Newly synthesized DNA in MRC5 cells was detected using the Cell-Light EdU DNA cell proliferation kit (RiboBio, Guangzhou, China) based on protocols. For CCK-8 analysis, MRC-5 cells (5000 cells/well) in 200 μl were plated into a 96-well microplate. After 20 μl of CCK8 (Beyotime, China) was added to each well for 1 h, the cell viability was detected at 450 nm following manual.

### Statistical analysis

All the data were presented by means ± SD, and all experiments were repeated at least three times. Independent-samples t-test was used to analyze two groups, and one-way analysis of variance (ANOVA) was used to analyze more groups with Dunnett’s test. *p* < 0.05 was considered significant.

## Results

### circZNF609 is downregulated in TGF-β1-induced activated fibroblasts and fibrotic lung tissues from silica-inhaled mice

**Fig. 1:**
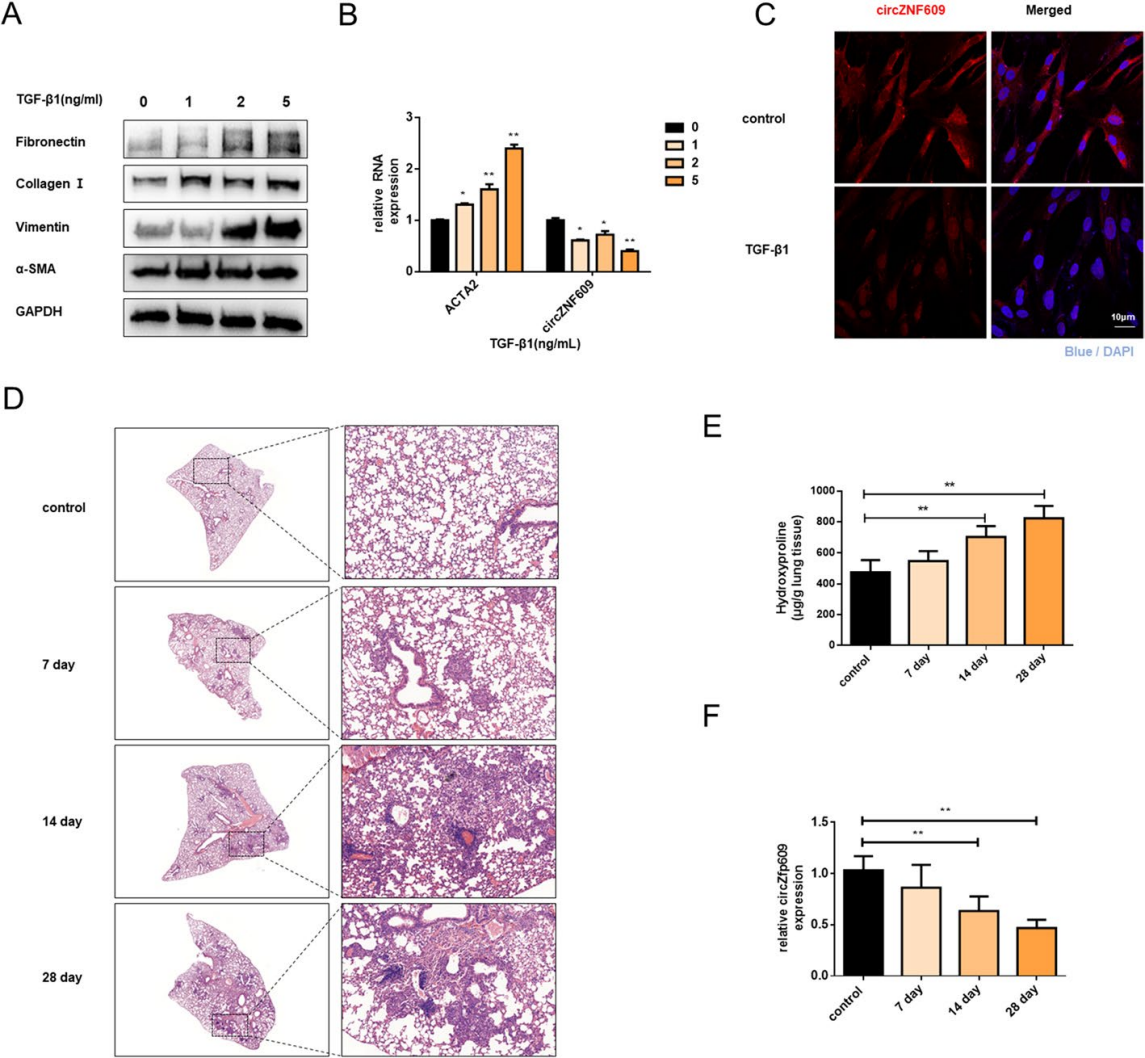
circZNF609 is downregulated in TGF-β1-induced activated fibroblasts and fibrotic lung tissues from silica-inhaled mice. **A** The protein levels of fibronectin, collagen I, vimentin, and α-SMA in each group were examined by the western blot. The results of the experiment were repeated at least three times. **B** The RNA expression of α-SMA and circZNF609 in MRC-5 cells using qRT-PCR analysis; GAPDH was used as the internal reference. **C** Fluorescence in situ hybridization (FISH) assay was conducted to detect the expression of circZNF609 in control and TGF-β1-treated groups. **D** Pathological changes in mouse lung tissues were presented by H&E staining, and the arrow pointed to the representative fibrosis foci (*n* = 6 for each group). **E** Hydroxyproline content of the lung tissues was used to examine the degree of collagen deposition. **F** qRT-PCR analysis of circZfP609 expression in mouse fibrotic lung tissues on days 7, 14, and 28 (*n* = 6 for each group). All data were expressed as the means ± SD of at least 3 independent experiments, *p < 0.05 and **p < 0.01

Though emerging studies have uncovered the pivotal function of circZNF609 in cancer [21, 22], its underlying role in pulmonary fibrosis remains elusive. To further confirm whether circZNF609 is related to lung fibrosis induced by silica, we started with the detection of the expression level of circZNF609. Based on the methods previously described [19], we added TGF-β1 in cell culture media to activate fibroblast. As expected, the protein levels of the mesenchymal cell marker (α-SMA, vimentin) and the extracellular matrix protein (Fibronectin, Collagen I) were obviously increased in a dose-dependent manner (Fig. 1A and Additional file 1: Figure S1A). Meanwhile, ACTA2 (actin alpha 2) was also upregulated while circZNF609 was significantly decreased (Fig. 1B). Consistently, the FISH assay also showed decreased induction of circZNF609 after TGF-β1 treatment (Fig. 1C and Additional file 1: Figure S1B).

Furtherly, we quantified the circZNF609 expression in silica-inhaled mouse lung tissue. H&E staining of silica-challenged lungs revealed the destruction of alveolar structure and typical fibrotic nodules along with the time after silica instillation (Fig. 1D). The hydroxyproline content assay (Fig. 1E) further verified the success of the mouse lung fibrosis model. Consistent with in vitro results, circZfp609 was significantly decreased in fibrotic mouse lung tissues (Fig. 1F).

### The characteristics of circZNF609

**Fig. 2:**
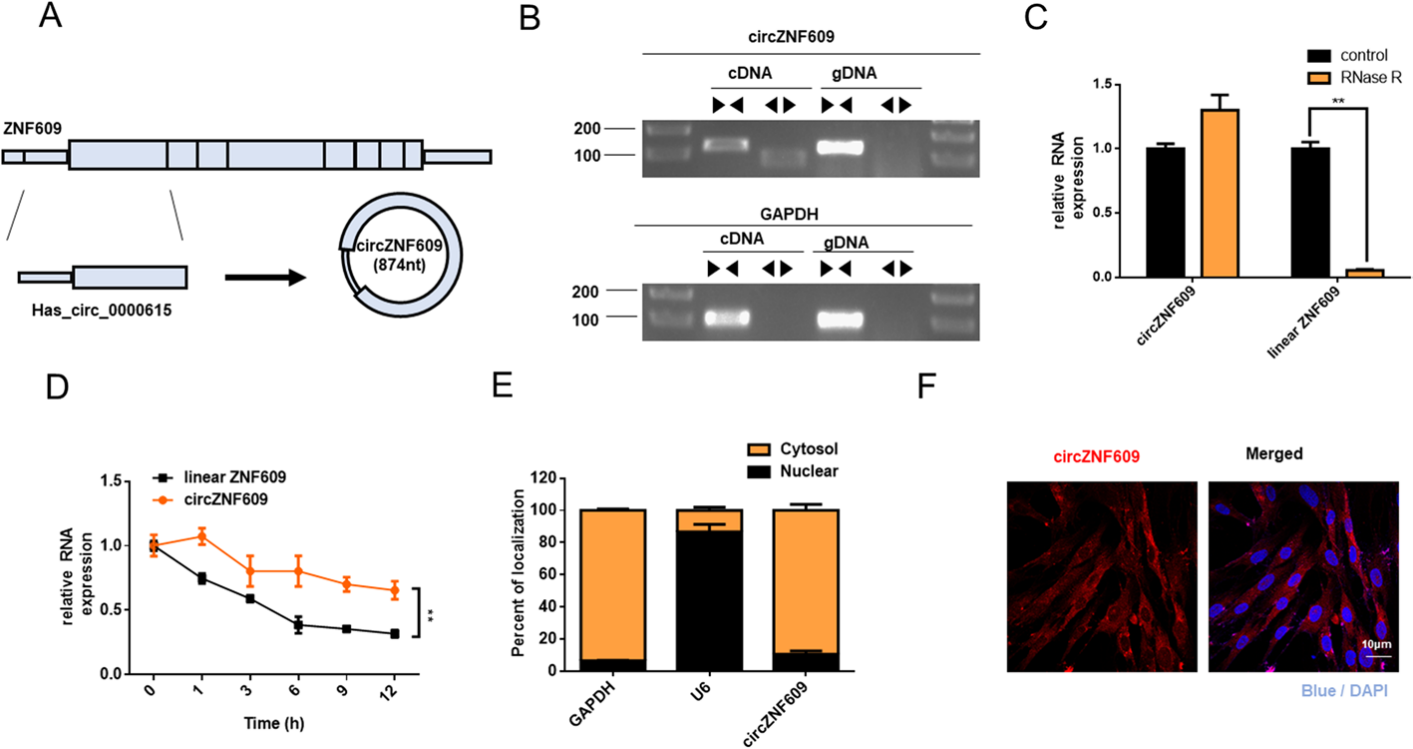
Characterization of the existence and subcellular distribution of circZNF609. **A** Schematic representation of the genomic location of circZNF609 together with its splicing pattern. **B** RT-PCR revealed that circZNF609 was amplified by divergent primers with cDNA, but not with genomic DNA (gDNA). GAPDH was used as a negative control. **C** The expression of circZNF609 and linear ZNF609 in MRC-5 was detected by qRT-PCR in the presence or absence of RNase R. **D** The RNA levels of circZNF609 and linear ZNF609 were analyzed by qRT-PCR in Actinomycin D-treated MRC-5 cells. **E** qRT-PCR analysis and FISH assay **F** were used to detect the expression of circZNF609 in the nuclear and cytoplasm of MRC-5. All data were expressed as the means ± SD of at least 3 independent experiments, *p < 0.05 and **p < 0.01

Before a functional study of circZNF609, we primarily testified the characteristics of the circZNF609 with loop structure (Fig. 2A). After examined by RT-PCR with divergent primers, circZNF609 could be detected in cDNA but not in gDNA, suggesting that circZNF609 was the product of tans-splicing instead of genomic rearrangements (Fig. 2B). Resistance to digestion by RNase R exonuclease further verified the circular RNA structure of circZNF609 (Fig. 2C). Following inhibition of transcription by Actinomycin D, the qRT-PCR analysis showed that circZNF609 was more stable and resistant than ZNF609 mRNA (Fig. 2D). We then evaluated the localization of circZNF609. Nuclear and cytoplasmic separation experiments (Fig. 2E) and FISH (Fig. 2F) assay demonstrated that circZNF609 was predominately distributed in the cytoplasm. Collectively, these results indicated that circZNF609 was enriched and stably expressed in fibroblasts.

### Overexpression of circZNF609 attenuates TGF-β1-indued fibroblast activation

**Fig. 3:**
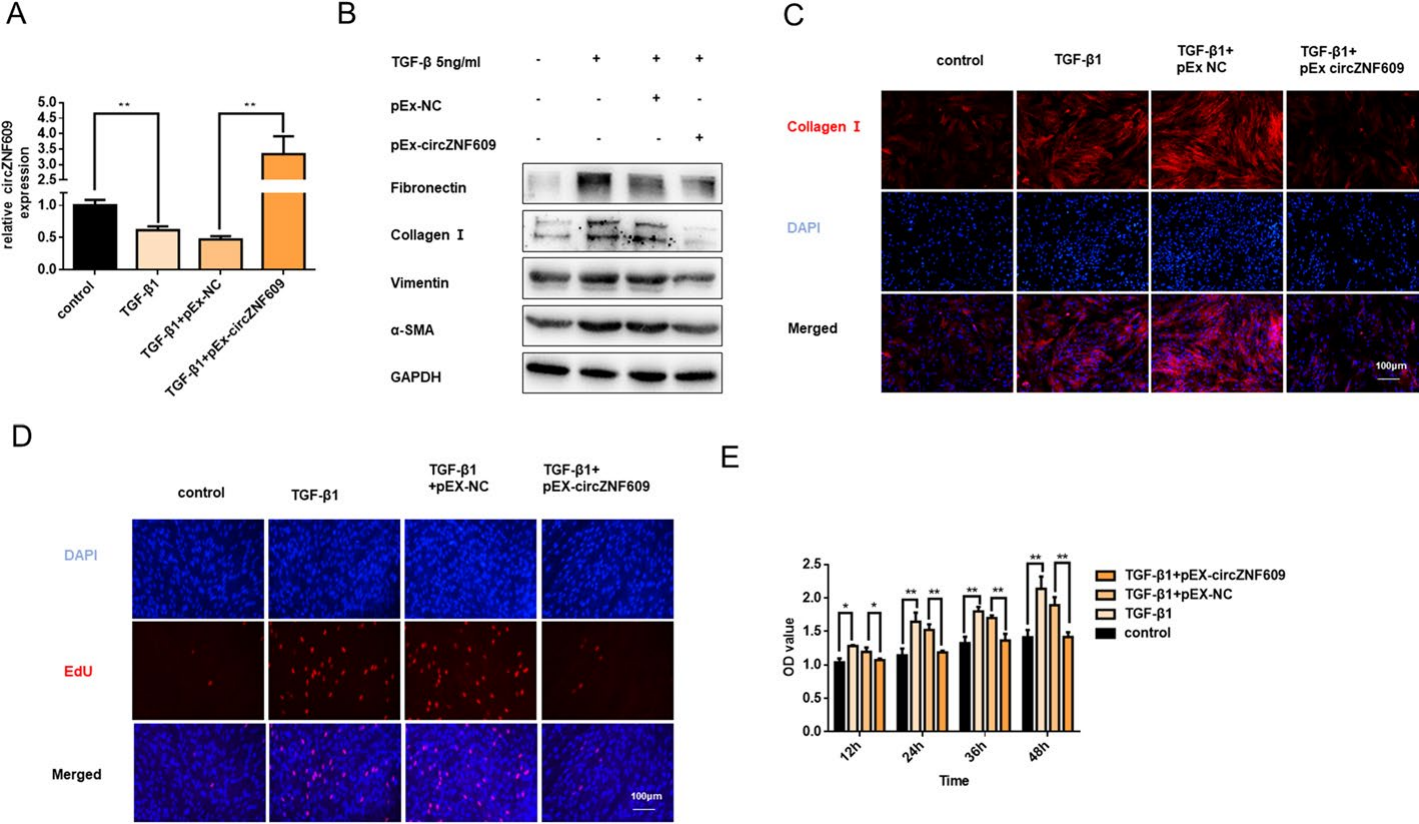
Overexpression of circZNF609 attenuates TGF-β1-indued fibroblast activation. **A** After being transfected with pEX-circZNF609 or pEX-NC, MRC-5 cells were administrated 5 ng/ml TGF-β1 for 48 h. The transfection efficiency was assessed via qRT-PCR analysis. **B** Western blotting showed fibronectin, collagen I, vimentin, and α-SMA protein levels. The results of the experiment were repeated at least three times. **C** Immunofluorescence staining detected α-SMA (red) levels in different groups; DNA staining by DAPI (blue) represents nuclear; bars = 100 μm. **D** EdU staining and CCK8 assay **E** for the assessment of cell proliferation in MRC-5 cells, showing that the overexpression of circZNF609 inhibited TGF-β1-induced cell viability and proliferation; bars = 100 μm. The data were expressed as the means ± SD of at least 3 independent experiments, *p < 0.05 and **p < 0.01

Given the significant downregulation of circZNF069 in activated fibroblasts and fibrotic mouse lung tissues, we further investigated the underlying function and molecular mechanism of circZNF609 in MRC-5 cell lines. To explore whether circZNF609 exerts anti-fibrotic roles in TGF-β1 stimulated fibroblasts, we constructed circZNF609 overexpression plasmid, and measured the transfection efficiency via qRT-PCR. As expected, compared with negative control, circZNF609 was upregulated in pEx-circZNF609-transfected cells (Fig. 3A). Furthermore, overexpression of circZNF609 inhibited the production of fibrotic markers (Fig. 3B), as verified by Immunofluorescence with the expression of αSMA (Fig. 3C). Consistently, Edu and CCK8 assay showed circZNF609 could restore TGF-β1-mediated fibroblast proliferation (Fig. 3D and E). Collectively, these findings suggest a major role of circZNF609 in fibroblast activation, and upregulated circZNF609 could rescue the fibroblast phenotype.

### circZNF609 inhibits pulmonary fibrosis progress via miR-145-5p/KLF4 axis

**Fig. 4:**
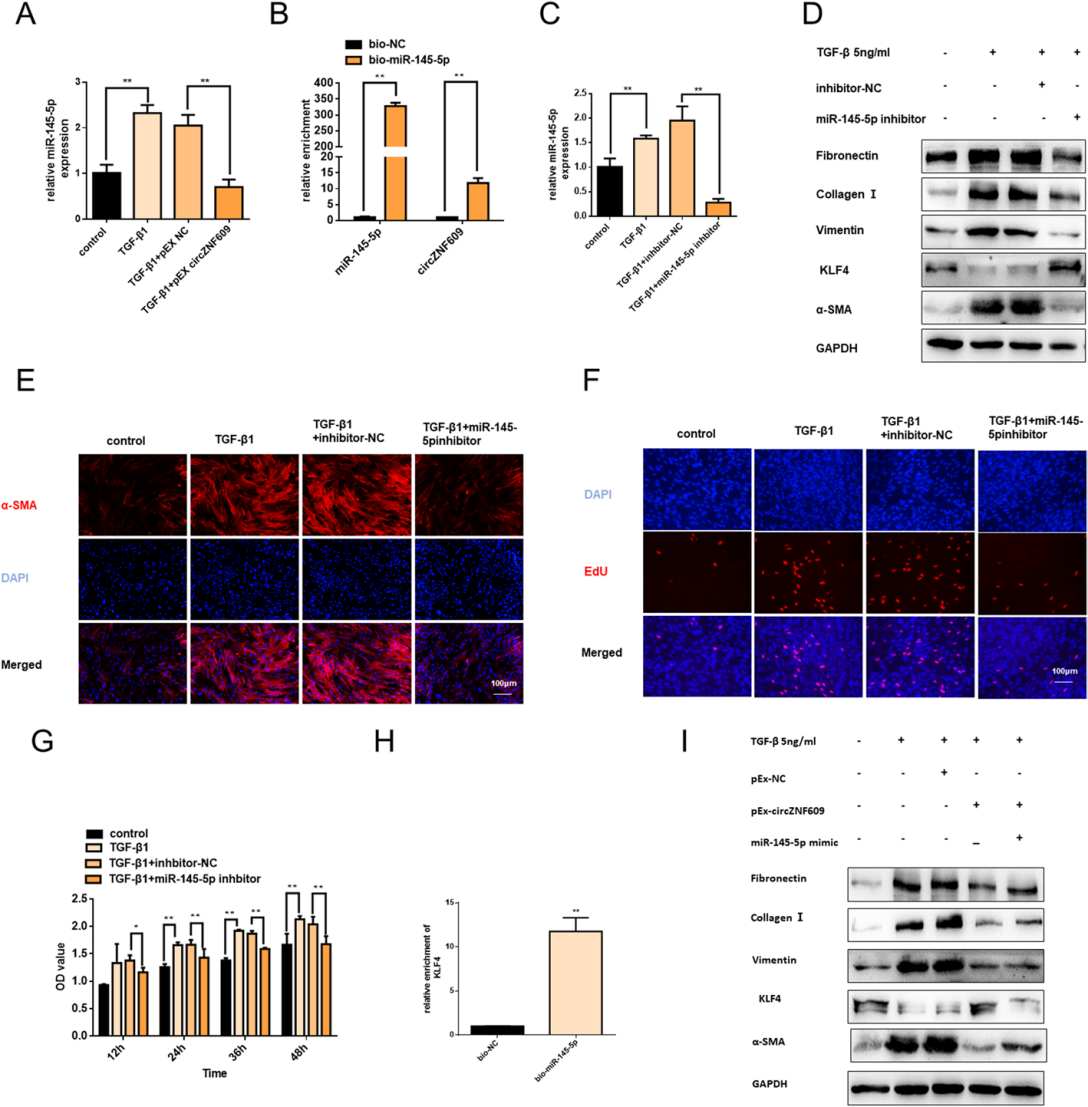
circZNF609 inhibits pulmonary fibrosis progress via miR-145-5p/KLF4 axis. **A** After being transfected with pEX-circZNF609 or pEX-NC, MRC-5 cells were administrated 5 ng/ml TGF-β1 for 48 h. The miR-145-5p level was testified via qRT-PCR analysis; U6 was used as the internal reference. **B** The interaction between miR-145-5p and circZNF609 was verified by RNA pulldown assay. **C** Transfection of miR-145-5p inhibitors significantly decreased miR-145-5p expression in MRC-5 cells treated with 5 ng/ml TGF-β1 for 48 h. **D** Western blotting showed fibronectin, collagen I, vimentin, KLF4, and α-SMA protein levels. The results of the experiment were repeated at least three times. **E** Immunofluorescence staining detected α-SMA (red) levels in different groups; DNA staining by DAPI (blue) represents nuclear; bars = 100 μm. **F** EdU staining and CCK8 assay **G** for the assessment of cell proliferation in MRC-5 cells, showing that the inhibition of miR-145-5p inhibited TGF-β1-induced cell viability and proliferation. **H** The interaction between miR-145-5p and KLF4 mRNA was verified by RNA pulldown assay. **I** After being co-transfected with pEX-circZNF609 and miR-145-5p inhibitors, MRC-5 cells were administrated 5 ng/ml TGF-β1 for 48 h. Western blotting showed fibronectin, collagen I, vimentin, KLF4, and α-SMA protein levels. All data were expressed as the means ± SD of at least 3 independent experiments, *p < 0.05 and **p < 0.01

Accumulating investigations have revealed that circRNAs can serve as microRNA sponges and subsequently eliminate the function of downstream target miRNAs. As we previously testified that circZNF609 was mainly distributed in the cytoplasm of MRC-5 cells and exhibited stability, which was a prerequisite for acting as a molecular sponge. Hence, a hypothesis was made that circZNF609 might be involved in TGF-β1-indued fibroblast activation by blocking target miRNAs. Three bioinformatics databases (circBank, starBase and Circular RNA Interactome) were used to predict underlying target miRNAs, and miR-145-5p was confirmed to be circZNF609 downstream miRNA after qRT-PCR detection. As the qRT-PCR analysis showed miR-145-5p was increased in TGF-β1-treated MRC-5 cells, but overexpressed circZNF609 could obviously inhibit miR-145-5p expression (Fig. 4A). Subsequent RNA pull-down assay and qRT-PCR result delineated that circZNF609 could be pulled down by biotin-labeled miR-145-5p, suggesting the combination between circZNF609 and miR-145-5p (Fig. 4B).

Now that we have confirmed that circZNF609 could directly target miR-145-5p, the roles of miR-145-5p in fibroblast activation would be investigated. As expected, the transfection of miR-145-5p inhibitor obviously inhibited the production of fibrotic markers (Fig. 4D, E and Additional file 3: Figure S3A and 3B). Edu and CCK8 assay showed inhibition of miR-145-5p could suppress TGF-β1-mediated fibroblast proliferation (Fig. 4F and G, Additional file 3: Figure S3C). Next, the online prediction tools (miRDB and TargetScan) were used to reveal the potential target genes of miR-145-5p, and we found Krueppel-like factor 4 (KLF4)— a well-known inhibitor of α-SMA. We conducted an RNA pull-down assay and found that endogenous KLF4 mRNA could be specifically pulled down by biotin-labeled miR-145-5p (Fig. 4H) in MRC-5 cells. Moreover, inhibition of miR-145-5p enhanced the protein level of KLF4 (Fig. 4D). To determine whether cricZNF609 attenuates TGF-β1-stimulated fibroblast activation via miR-145-5p/KLF4 axis, we co-transfected pEx-circZNF609 and miR-145-5p mimics. Upregulation of miR-145-5p partly reversed the circZNF609-overexpression-induced antifibrotic effect (Fig. 4I and Additional file 3: Figure S3D). Together, these data suggest that circZNF609 regulates fibroblast activation via miR-145-5p/KLF4 axis.

### Overexpression of circZNF609 inhibits silica-induced mouse pulmonary fibrosis in vivo

**Fig. 5:**
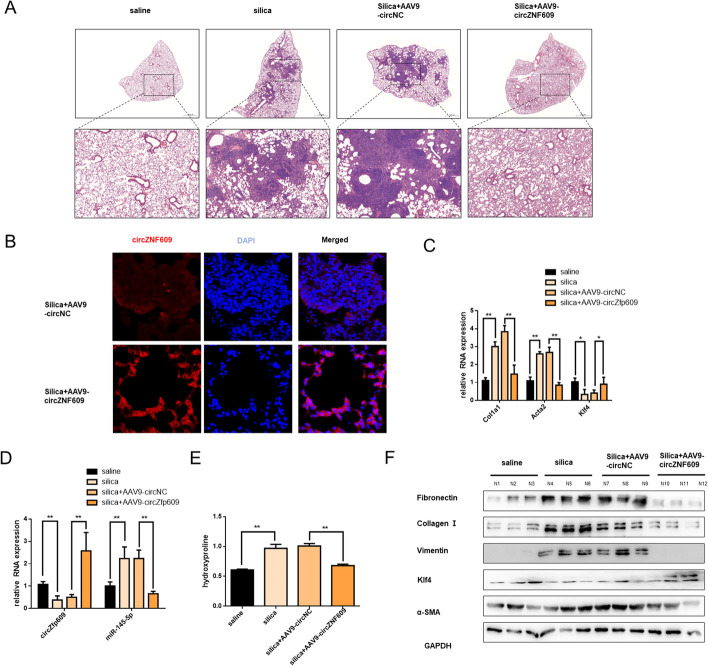
Overexpression of circZNF609 inhibits silica-induced mouse pulmonary fibrosis in vivo. **A** Sections stained with H&E suggested the lung fibrotic lesion of each group, and the arrow pointed to the representative fibrosis foci (*n* = 6 in each group). **B** FISH assay was conducted to detect the expression of circZfp609 in silica + AAV9-NC and silica + AAV9-circZfp609 groups. **C** and **D** qRT-PCR analysis of Col1a1, Acta2, Klf4, circZfp609 and miR-145-5p expression in mouse lung tissues. **E** The collagen deposition was detected by a hydroxyproline content assay. **F** Western blot analysis of fibronectin, collagen I, vimentin, klf4 and α-SMA in each group. The results of the experiment were repeated at least three times. All data were expressed as the means ± SD of at least 3 independent experiments, *p < 0.05 and **p < 0.01

To further examine the effect of circZNF609 on lung fibrosis in vivo, we increased circZfp609 expression in mouse lung tissues via intratracheal instillation AAV9-circZfp609. As expected, overexpression of circZfp609 alleviated the fibrosis (Fig. 5A). Fish assay (Fig. 5B) showed that AAV9-circZfp609 instillation increased the circZfp609 expression in mouse lung tissue. Consistent with this, qRT-PCR results showed that circZfp609 upregulation obviously decreased the expression of Col1a1, Acta2, Klf4 and miR-145-5p genes (Fig. 5C and D). Moreover, Hydroxyproline and western blot assays showed enhanced expression of circZfp609 relieved silica-induced pulmonary fibrosis and collagen deposition (Fig. 5E and F, and Additional file 4: Figure S4A). To sum up, these experimental results suggested the antifibrotic effect of circZfp609 in silica-treated mouse lung tissues via miR-145-5p/Klf4 axis.

### m6A regulates circZNF609

**Fig. 6:**
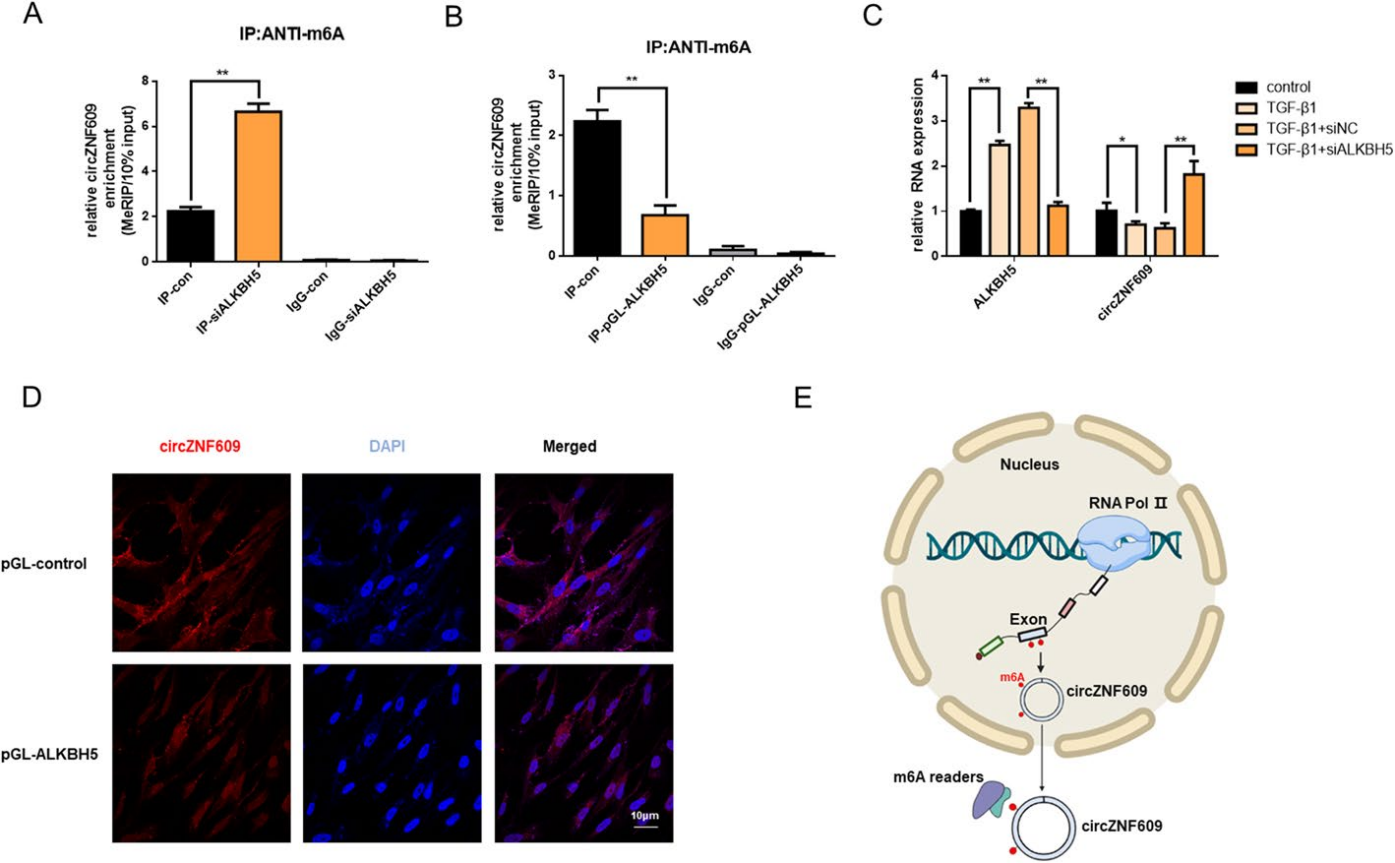
m6A regulates circZNF609. **A** The detection of circZNF609 m6A modification levels by RIP of m6A modified circRNA in control or siALKBH5 groups followed by qRT-PCR. **B** The detection of circZNF609 m6A modification levels by RIP of m6A modified circRNA in control or pGL-ALKBH5 groups followed by qRT-PCR. **C** qRT-PCR analysis of ALKBH5 and circZNF609 expression in each group. **D** The FISH assay was conducted to determine the subcellular localization and expression of circZNF609 in control and pGL-ALKBH5 groups. **E** Schematic representation of the biogenesis and export of circZNF609 regulated by m6A modification. All data were expressed as the means ± SD of at least 3 independent experiments, *p < 0.05 and **p < 0.01

CircZNF609 was reported to be generated by precursor mRNA post-splicing of exon 2 of the ZNF609 transcript. However, the specific molecular mechanisms controlling circZNF609 levels remain intricate. M6A is an important modification in RNA, which may be involved in circRNA biogenesis. Previously, we found AlkB homolog 5 (ALKBH5), a m6A demethylase, was elevated in the TGF-β1 treated fibroblast [19]. Importantly, m6A-specific RIP assays revealed that ALKBH5 knockdown increased the amount of circZNF609 modified by m6A, while ALKBH5 overexpression decreased the m6A modification (Fig. 6A and B). Moreover, we found that knockdown ALKBH5 obviously increased the expression of circZNF609 (Fig. 6C). In addition, overexpression of ALKBH5 inhibited the cytoplasmic output of circZNF609 as FISH assay showed (Fig. 6D). These data indicated that m6A modification could control the biogenesis and export of circZNF609 (Fig. 6E).

### circZNF609 encodes peptides

**Fig. 7:**
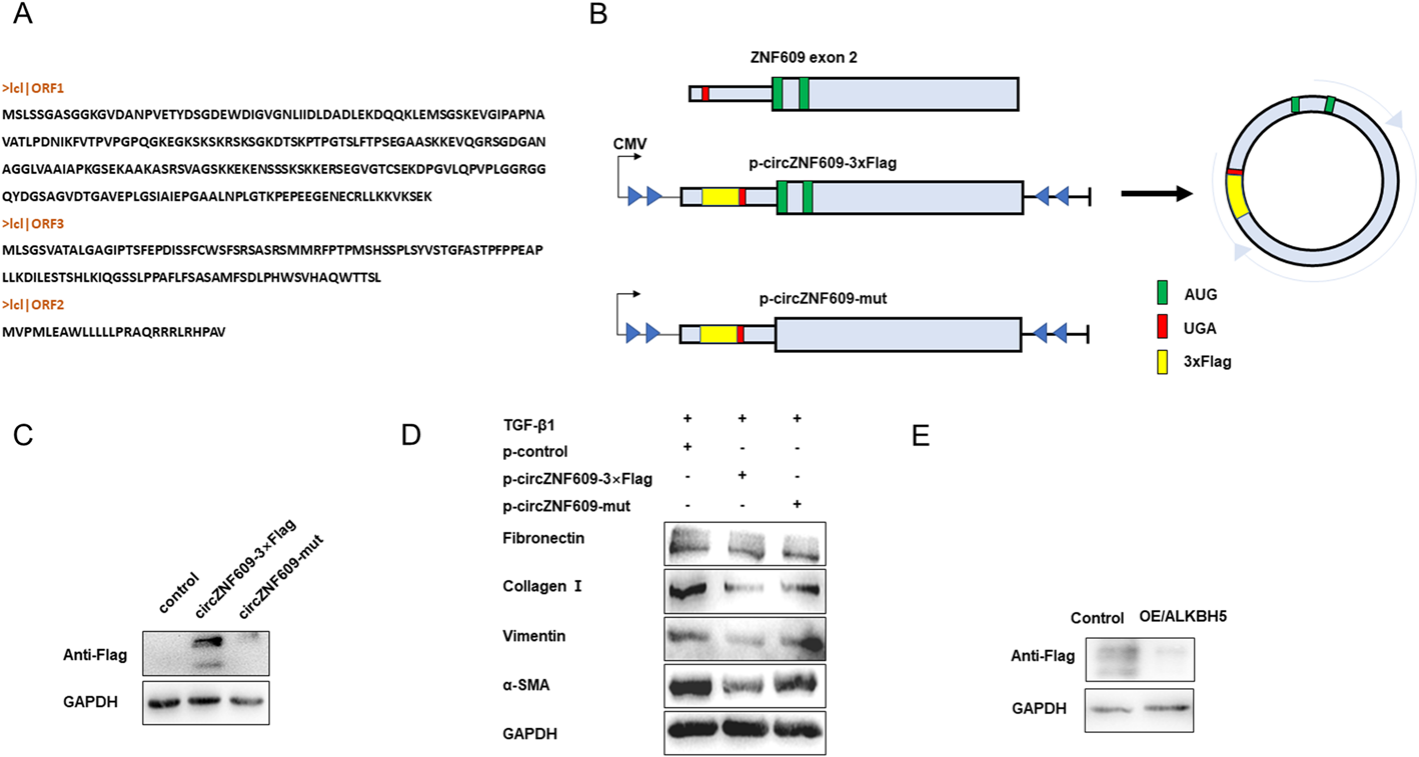
circZNF609 encodes peptides. **A** The predicted sequence of circZZNF609-derived peptides. **B** Schematic representation of the p-circZNF609-3xFlag construct and the corresponding circRNA. Start and stop codons are shown in green and red, respectively. A 3 × FLAG-coding sequence is shown in yellow. **C** Representative western blots of the proteins derived from circZNF609-3xFlag construct. The results of the experiment were repeated at least three times. **D** Western blot analysis of fibronectin, collagen I, vimentin, and α-SMA in each group. The results of the experiment were repeated at least three times. **E** Representative western blots of the proteins derived from circZNF609-3xFlag construct upon ALKBH5 overexpression. The results of the experiment were repeated at least three times

Emerging evidence suggests that circRNAs can encode proteins, and proteins translated from circRNAs can regulate multiple biological functions [23–26]. In 2017, Ivano and colleagues verified that a portion of the circZNF609 could load onto heavy polysome fractions via sucrose gradient fractionation [26], indicating that circZNF609 has protein-coding activity. Three open reading frames (ORF) and relative amino acid sequence were predicted by the ORFfinder (https://www.ncbi.nlm.nih.gov/orffinder/), as shown in Fig. 7A. To confirm the encoding capacity of circZNF609 in lung fibroblast, we constructed the overexpression plasmids of circZNF609 and its start codon mutant plasmid (Fig. 7B). There is 3xFlag-coding sequence upstream of the Stop codon in these plasmids, which could generate a flagged protein when the circular template is formed. After transfected p-circZNF609-3xFlag, flagged peptides could be observed, but not in the p-circZNF609-mut group (Fig. 7C and Additional file 5: Figure S5A). We further detected the indicators of fibrosis and found that p-circZNF609-3xFlag partially inhibit the fibroblast activation (Fig. 7D and Additional file 5: Figure S5B). Due to the known role of m6A in circZNF609, we tested the effects of ALKBH5 on the translation of circZNF609 derived from the p-circZNF609-3xFlag. The overexpression of ALKBH5 decreased the level of flagged peptides (Fig. 7E and Additional file 5: Figure S5C).

## Discussion

The development of silica-induced pulmonary fibrosis through fibroblast activation is extraordinarily complex, involving multiple molecules and mechanisms. A thorough understanding of the molecular basis of uncontrolled fibroblast activation is indispensable to identify promising anti-fibrosis therapy targets. This study aimed to reveal the function of circZNF609 in silica-induced lung fibrosis. We found that circZNF609 was downregulated in silica-inhaled mouse lung tissue and TGF-β1-treated activated fibroblasts, suggesting the underlying clinical relevance of circZNF609 in lung fibrosis. Overexpression of circZNF609 could ameliorate TGF-β1-induced fibroblast-to-myofibroblast transition and attenuate lung fibrosis in silica-inhaled mice.

An increasing number of studies have suggested that endogenous circRNAs play a critical role in the development of various diseases, such as atherogenesis, Parkinson’s disease, and various cancers [27–29]. Compared to linear RNAs, circRNAs have a longer half-life and are more resistant to RNase R due to their covalently closed circular structure [30], making them promising candidates for early detection markers and novel therapeutic targets. In patients with hypertrophic cardiomyopathy (HCM), serum levels of circTMEM56 and circDNAJC6 were significantly different between healthy and HCM patients, emerging as viable biomarkers for HCM aid in the clinical decision making [31]. Previous studies have shown that circZNF609 is involved in the proliferative and migratory ability of lung cancer cells [32, 33]. However, the function of cricZNF609 in fibrotic diseases remains unclear, let alone in pulmonary fibrosis. In the present study, we revealed the expression pattern of circZNF609 in silica-induced fibrotic mouse lung tissue and TGF-β1-treated fibroblasts. The activation of pulmonary fibroblast is central to the pathogenesis of lung fibrosis; however, the mechanism driving fibroblast activation remains elusive. We used TGF-β1-treated MRC-5 cells to mimic fibroblast activation in silicosis. MRC-5 cells are embryonic lung cells with fibroblast characteristics. Consistent with fibroblasts in the lung tissue of silicosis patients, MRC-5 cells proliferate and activate and secrete excessive ECM after treatment with TGF-β1 and are widely used in pulmonary fibrosis studies. TGF-β1 is widely recognized as the most potent fibrogenic factor in fibrosis, and our previous studies did not find direct effects of silica on fibroblast activation, making that TGF-β1 was chosen to induce fibroblast activation. In this study, we demonstrated that enhanced expression of circZNF609 suppressed fibroblast activation by TGF-β1 treatment, suggesting that circZNF609 may serve as a fibrosis inhibitor in silica-induced lung fibrosis via regulating fibroblast activation.

The majority of circRNAs are found predominantly in the cytoplasm [34], an observation that prompted investigators to explore the role of circRNAs in sequestering miRNAs by acting as a sponge. Previously, circZNF609 was shown to promote the progression of hepatocellular carcinoma by sponging miR-15a-5p/15b-5p [35]. According to the prediction of bioinformatics tools, we screened and experimentally verified that miR-145-5p could interact with circZNF609. Moreover, miR-145-5p has been revealed that play an important function in the differentiation of lung myofibroblasts by targeting KLF4 [36]. Here, we found that overexpression of circZNF609 inhibited lung fibroblast activation via the miR-145-5p/KLF4 axis. However, as a pluripotency transcription factor, the role of KLF4 in myofibroblast differentiation and fibrosis remains controversial. For instance, KLF4 initiates sustained YAP activation and high expression to promote renal fibrosis [37], which is the opposite of lung fibrosis. Intriguingly, a recent report demonstrated that KLF4 is a vital regulator in the lung mesenchyme during fibrosis, with opposite cell type-specific effects: profibrotic in PDGFR-β + cells and antifibrotic in SMA + cells [38]. The cell type-specific functions of KLF4 may underlie the distinct roles of KLF4 in fibrosis. In bleomycin-induced pulmonary fibrosis, KLF4 was downregulated in the fibrotic area, and KLF4 overexpression attenuated bleomycin-induced pulmonary fibrosis [39], consistent with our observations. Thus, we hypothesized that although KLF4 has opposing cell type-specific roles in lung fibrosis, changes in the pulmonary environment caused by silica dust or bleomycin led to the transformation of PDGFR-β + cells into SMA + myofibroblasts. A previous finding supports our hypothesis, which confirmed that human pericytes generate α-SMA + cell populations in response to changes in the fibrotic lung microenvironment [40].

Although circRNAs have long been thought to function as non-coding RNAs, emerging evidence has showed that circRNAs can encode peptides, providing a new perspective for studying the role of circRNAs in disease [24]. Exploring the translational function of circRNAs will reveal a hidden proteome and enhance our understanding of the importance of circRNAs in human disease. For example, circβ-catenin could encode a novel β-catenin isoform, called β-catenin-370aa, which competitively interacts with GSK3β and promotes tumour growth via the Wnt/β-catenin pathway in liver cancer [23]. CircMAPK1 plays a tumour suppressor role in gastric cancer via its encoded protein MAPK1-109aa [41]. These findings reveal a non-canonical function of circRNAs and further highlight the critical roles of circRNAs in regulating diverse physiological and pathological processes. However, though previous studies have demonstrated that circZNF609 contains a 753-nt open reading frame (ORF) and the protein coding ability [26], no studies have explored the physiological and pathological functions of the circZNF609-derived protein. In the current study, we found that the peptides encoded by circZNF609 could partially attenuate the activation of MRC-5 cells. As a member of the zinc finger protein family, ZNF609 is characterized by two zinc finger protein domains. However, the peptides encoded by circZNF609 lack the infringer domains, suggesting that it may function by a different mechanism than the full-length isoform, such as acting as a dominant-negative competitor. Overall, our data show that a novel protein encoded by circRNA provides a promising potential therapeutic target for the treatment of pulmonary fibrosis.

To date, two potential mechanisms have been proposed to drive circRNA translation—internal Ribosome Entry site (IRES)- and N6-methyladenosine (m6A)-mediated translation initiation [42]. Interestingly, it has been reported that circZNF609 can not only drive IRES-dependent translation [26], but also can drive (m6A)-mediated translation [43]. Our previous study revealed that AlkB homolog 5 (ALKBH5) is involved in regulating silica-induced pulmonary fibrosis [19]. In this study, we further demonstrated that ALKBH5 could control circZNF609 expression and cytoplasmic export in an m6A-dependent manner. In addition, we observed that ALKBH5 knockdown reduced the levels of the peptides encoded by circZNF609, which is similar to the results of a previous study [43]. Since ALKBH5 could control the circZNF609 biogenesis and cytoplasmic export in an m6A-dependent manner, it is difficult to determine whether the change in peptide levels is caused by controlling the circZNF609 biogenesis or by controlling the translation ability. This also indicate the complexity, variability, and importance of the role of m6A modification.

In conclusion, we revealed that cricZNF609 is a critical regulator of fibroblast activation and silica-induced lung fibrosis. This is valuable because there is an urgent need to develop novel agents for fibrosis treatment. In the present study, we demonstrated that cricZNF609 is highly methylated and that this modification regulates circZNF609 biogenesis and cytoplasmic export. Subsequent mechanistic exploration revealed that overexpression of circZNF609 could inhibit fibroblast activation via the miR-145-5p/KLF4 axis. Simultaneously, we also found that circZNF609-derived peptides exerted anti-fibrotic effects in MRC-5 cells, indicating that peptides encoded from circRNAs may be potential pharmacological targets for the treatment of lung fibrosis. Our study is not free of limitations; for example, we revealed that circZNF609 may function by acting as a sponge and encoding peptides in pulmonary fibrosis, but it is not clear which one dominates. It is not uncommon for key regulators to function through multiple pathways. Nonetheless, the sum of our findings highlights cricZNF609 as a promising target for anti-pulmonary fibrosis strategies.

## Conclusions

The present findings suggest that circZNF609 is a critical regulatory factor in fibroblast activation and silica-induced lung fibrosis. The circZNF609 and its derived peptides may provide novel promising targets for the treatment of pulmonary fibrosis.

### Supplementary Information

**Additional file 1: Figure S1.** (**A**) Quantification of immunoblots in Fig. 1A. (**B**) Quantification of fluorescence intensity in Fig. 1C.

**Additional file 2: Figure S2.** (**A**) Quantification of immunoblots in Fig. 3B. (**B**) Quantification of fluorescence intensity in Fig. 3C. (**C**) Quantification of relative number of EdU+ cells in Fig. 3D.

**Additional file 3: Figure S3.** (**A**) Quantification of immunoblots in Fig. 4D. (**B**) Quantification of fluorescence intensity in Fig. 4E. (**C**) Quantification of relative number of EdU+ cells in Fig. 4F. (**D**) Quantification of immunoblots in Fig. 4I.

**Additional file 4.** (**A**) Quantification of immunoblots in Fig. 5F.

**Additional file 5: Figure S5.** (**A**) Quantification of immunoblots in Fig. 7C. (**B**) Quantification of immunoblots in Fig. 7D. (**C**) Quantification of immunoblots in Fig. 7E.

## Data Availability

The data from this study are available in this published article.

## Abbreviations

circZNF609: Circular ZNF609
KLF4: Krueppel-like factor 4
TGF-β1: Transforming growth factor-β1
m6A: N6-methyladenosine
RIP: RNA immunoprecipitation
α-SMA: Alpha-smooth muscle actin
ECM: Extracellular matrix
miRNA: MicroRNA
qRT-PCR: Quantitative real-time PCR
siRNA: Small interfering RNA
IPF: Idiopathic pulmonary fibrosis
H&E: Hematoxylin and eosin
H&E: Hematoxylin and eosin
FISH: Fluorescence in situ hybridization assay
ORF: Open reading frames
HCM: Hypertrophic cardiomyopathy
IRES: Internal ribosome entry site
ALKBH5: AlkB homolog 5
