# Supplementary material for: CircZNF609 regulates pulmonary fibrosis via miR-145-5p/KLF4 axis and its translation function
Source: Cell Mol Biol Lett. 2023 Dec 18;28:105. doi: 10.1186/s11658-023-00518-w (PMC10726587; doi:10.1186/s11658-023-00518-w)
Supplement: Supplementary file 1 — Additional file 1: Figure S1. (A) Quantification of immunoblots in Fig. 1A. (B) Quantification of fluorescence intensity in Fig. 1C. [file 11658_2023_518_MOESM1_ESM.docx]

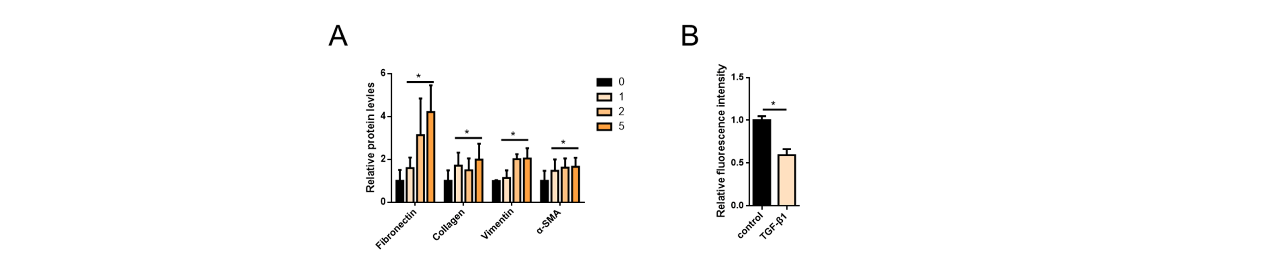


**Figure S1. (A) Quantification of immunoblots in Figure 1A. (B) Quantification of fluorescence intensity in Figure 1C.**
