# Supplementary material for: CircZNF609 regulates pulmonary fibrosis via miR-145-5p/KLF4 axis and its translation function
Source: Cell Mol Biol Lett. 2023 Dec 18;28:105. doi: 10.1186/s11658-023-00518-w (PMC10726587; doi:10.1186/s11658-023-00518-w)
Supplement: Supplementary file 3 — Additional file 3: Figure S3. (A) Quantification of immunoblots in Fig. 4D. (B) Quantification of fluorescence intensity in Fig. 4E. (C) Quantification of relative number of EdU+ cells in Fig. 4F. (D) Quantification of immunoblots in Fig. 4I. [file 11658_2023_518_MOESM3_ESM.docx]

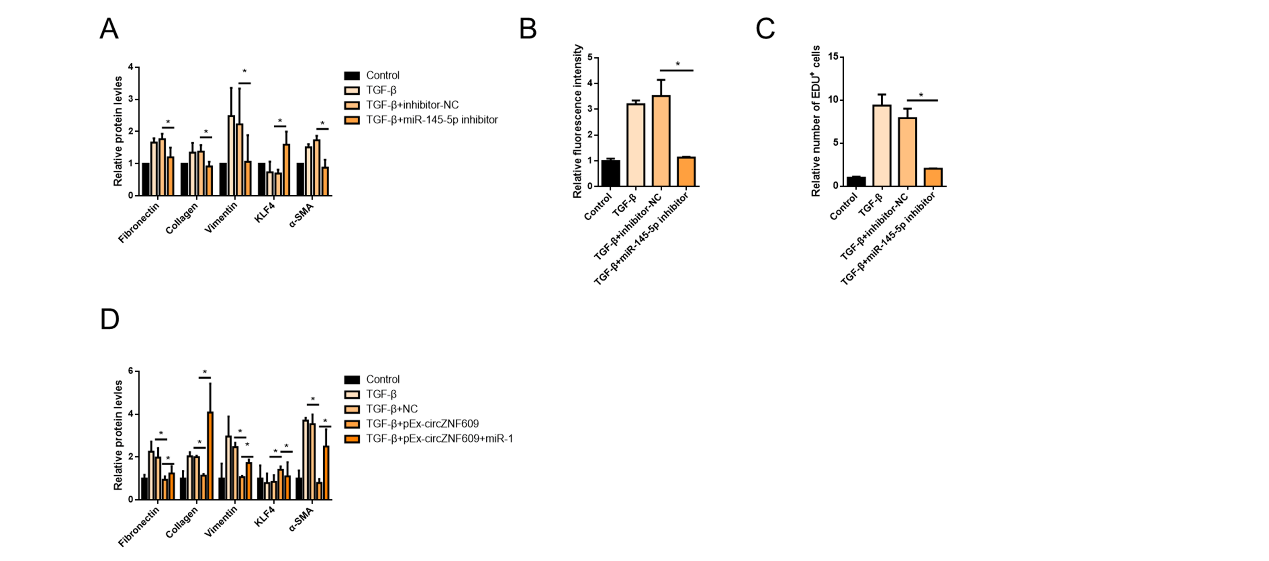


**Figure S3. (A) Quantification of immunoblots in Figure 4D. (B) Quantification of fluorescence intensity in Figure 4E. (C) Quantification of relative number of EdU^+^ cells in Figure 4F. (D) Quantification of immunoblots in Figure 4I.**
