# Supplementary figures and images for: CircZNF609 regulates pulmonary fibrosis via miR-145-5p/KLF4 axis and its translation function
Source: Cell Mol Biol Lett. 2023 Dec 18;28:105. doi: 10.1186/s11658-023-00518-w (PMC10726587; doi:10.1186/s11658-023-00518-w)

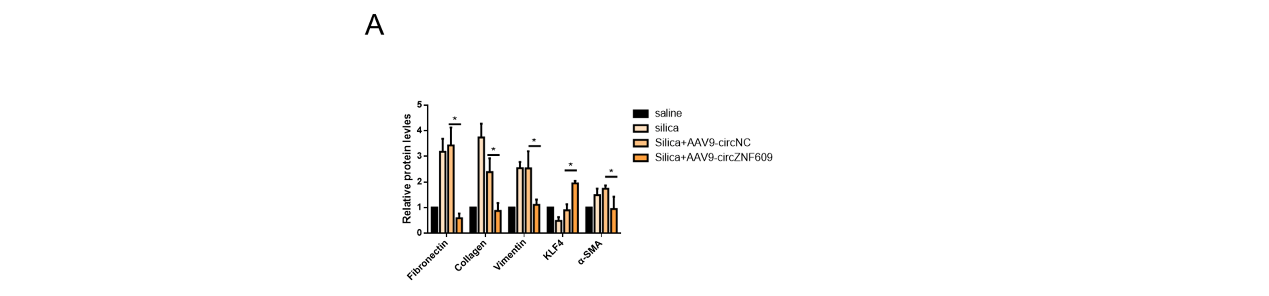


**Figure S4. (A) Quantification of immunoblots in Figure 5F.**

Supplement: Supplementary file 4 — Additional file 4. (A) Quantification of immunoblots in Fig. 5F. [file 11658_2023_518_MOESM4_ESM.docx]
