# Supplementary material for: CircZNF609 regulates pulmonary fibrosis via miR-145-5p/KLF4 axis and its translation function
Source: Cell Mol Biol Lett. 2023 Dec 18;28:105. doi: 10.1186/s11658-023-00518-w (PMC10726587; doi:10.1186/s11658-023-00518-w)
Supplement: Supplementary file 5 — Additional file 5: Figure S5. (A) Quantification of immunoblots in Fig. 7C. (B) Quantification of immunoblots in Fig. 7D. (C) Quantification of immunoblots in Fig. 7E. [file 11658_2023_518_MOESM5_ESM.docx]

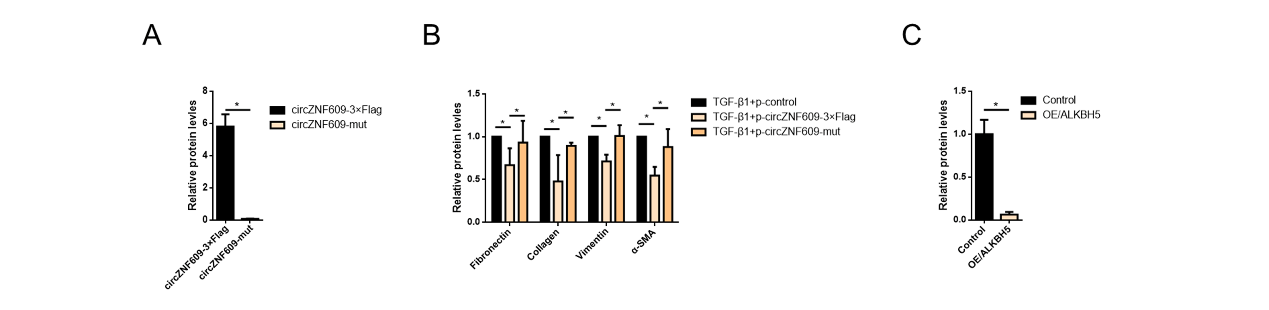


**Figure S5. (A) Quantification of immunoblots in Figure 7C. (B) Quantification of immunoblots in Figure 7D. (E) Quantification of immunoblots in Figure 7E.**
